# Electroacupuncture with or without combined warm needling for tinnitus: Study protocol for a randomized, waitlist-controlled trial

**DOI:** 10.1097/MD.0000000000034315

**Published:** 2023-07-21

**Authors:** Yuanqi Guo, Lai Fun Ho, Liyi Chen, Ping Him Tsang, Min Chen, Kam Leung Chan, Bacon Fung-Leung Ng, Zhi Xiu Lin

**Affiliations:** a Chinese Medicine Services, Pok Oi Hospital, Hong Kong SAR, China; b School of Chinese Medicine, Faculty of Medicine, The Chinese University of Hong Kong, Shatin, NT, Hong Kong SAR, China; c Hong Kong Institute of Integrative Medicine, Faculty of Medicine, The Chinese University of Hong Kong, Shatin, NT, Hong Kong SAR, China; d Chinese Medicine Department, Hospital Authority, Kowloon, Hong Kong SAR, China.

**Keywords:** acupuncture, electroacupuncture, randomized controlled trial, study protocol, tinnitus, warm needling

## Abstract

Tinnitus is a common auditory condition that can lead to serious problems. Clinically, acupuncture and moxibustion have been commonly applied to treat tinnitus in China, with potential therapeutic effects but with limitations in study methodology and high-quality evidence. Therefore, we designed a randomized controlled trial to evaluate the efficacy and safety of either electroacupuncture alone or combined with warm needling for reducing tinnitus loudness and improving quality of life.

**Methods:**

This study is a prospective, multicenter, assessor-blind, 3-arm, parallel-group, randomized, waitlist-controlled trial. In total, 90 patients will be randomly assigned to the electroacupuncture, electroacupuncture and warm needing, or waitlist control group in a 1:1:1 ratio. Patients in the 2 treatment groups will be treated twice a week for a total of 5 weeks. Patients in the control group will not receive treatment during the study period and will be informed that they can receive it for free after a 10-week waiting period. The duration of intervention for this study will be 5 weeks, followed by another 5 weeks for the posttreatment assessment. The primary outcome is the change in the visual analog scale score for tinnitus loudness from baseline until the end of treatment. The secondary outcome is the tinnitus discomfort assessment measured using the Tinnitus Handicap Inventory. Outcome parameters will be assessed at baseline and at weeks 5 and 10. Any adverse events will be observed and recorded for safety assessment. Linear mixed models for repeated measures will be applied in the analysis.

**Discussion:**

Acupuncture and moxibustion could be potentially effective treatment alternatives for tinnitus. The study results will provide evidence to determine the efficacy and safety of electroacupuncture with or without warm needling for tinnitus.

## 1. Introduction

Tinnitus is a common auditory condition in otolaryngology practice. It refers to the perception of sound in the ear or brain, in the absence of external auditory stimulation.^[1–3]^ People usually experience an auditory phantom sensation even when no external sound is present. It can be any sound but is typically characterized as a ringing, hissing, buzzing, whistling, or roaring sound heard in 1 or both ears, or somewhere inside the head.^[1]^

Epidemiological studies report a wide range prevalence of tinnitus in adults ranging from 5.1% to 42.7%.^[4]^ In China, the prevalence of tinnitus ranges from 4.3% to 51.3%; however, it varied with age and sex.^[5]^

The pathophysiological mechanism of tinnitus is complex and not well-defined.^[6]^ While many people with tinnitus are able to manage it, it can also become a significant issue and may have a major impact on daily life, potentially leading to suicidal thoughts and attempts.

Previous studies indicated that the prevalence of tinnitus increases with age.^[5–7]^ Among older adults, 1 study showed that 1 in 5 persons has tinnitus and every 1 out of 10 with tinnitus experienced severe tinnitus that interferes with daily life, and those with hearing impairment were twice as likely to have tinnitus compared to those without.^[8]^ Additionally, the prevalence and risk of developing tinnitus are associated with sex, hearing loss, otological comorbidity, systemic diseases, sleep disorders, stress, depression, and smoking.^[5–7]^

Various treatment approaches are currently used in clinical settings with varying levels of evidence, including cognitive behavioral therapy, tinnitus retraining therapy, counseling, hearing aids, cochlear implant therapy, and drug therapy.^[1,2,6]^ However, the best way to manage tinnitus remains inconclusive, and the methodological quality of studies varies. In addition, no specific therapy has been proven to be effective in completely eliminating tinnitus.

Acupuncture, one of the treatment modalities in traditional Chinese medicine (TCM) for tinnitus,^[9]^ has been widely used and recommended for tinnitus relief in China for a long time.^[10]^ The use of acupuncture for tinnitus was initially discussed in the Huangdi Neijing. Acupuncture and moxibustion can help relieve tinnitus by regulating the flow of blood and *qi*.^[10]^ Previous systematic reviews have shown promising results for manual acupuncture, electroacupuncture, or warm needling in reducing the loudness and disability of tinnitus.^[11–13]^ A Bayesian analysis showed that there was a trend of greater effectiveness of warm needling in the treatment of neurological tinnitus,^[14]^ but with limitations in study methodology and evidence.

To date, there is a lack of rigorous evidence for effective therapies to reduce or eliminate tinnitus. Further research on the therapeutic effects of acupuncture for tinnitus is warranted, and should be conducted according to higher methodological standards. Finally, given the importance of the health problem of tinnitus, a well-designed randomized controlled clinical trial is proposed.

Therefore, this study aims to explore and determine the efficacy and safety of electroacupuncture (EA) and electroacupuncture combined with warm needling (EAWN) for the treatment of subjective tinnitus among older adults in Hong Kong, and report the short-term follow-up results. We will compare the effects of the 2 acupuncture treatment groups and the control group in reducing the loudness of tinnitus and improving the impairment caused by tinnitus.

## 2. Methods

### 2.1. Study design and setting

This study is a prospective, multicenter, assessor-blind, triple-arm, parallel-group, randomized controlled trial with a 1:1:1 ratio of group allocation to EA, EAWN, or waitlist control (control group, who can receive acupuncture treatment after a 10-week waiting period). Ninety eligible participants will be recruited for this study. The entire study period will last for 10 weeks, including a 5-week intervention period and a 5-week follow-up period. Outcome assessments will be performed at baseline and at weeks 5 and 10 through telephone or face-to-face evaluations. The study will be conducted at 3 Chinese medicine clinics under the management of Pok Oi Hospital, namely, Pok Oi Hospital − The Chinese University of Hong Kong Chinese Medicine Clinic cum Training and Research Centre (Shatin District), Pok Oi Hospital − The Chinese University of Hong Kong Chinese Medicine Clinic cum Training and Research Centre (Yuen Long District), and Pok Oi Hospital − Chinese Medicine Polyclinic (Tin Shui Wai). The study protocol is written following the Standard Protocol Items: Recommendations for Interventional Trials 2013 Statement and the Standard Protocol Items for Clinical Trials with TCM 2018 checklists.^[15,16]^

### 2.2. Study population

This study will focus on subjects aged between 50 and 70 years with tinnitus for half a month to 24-month duration. Eligibility criteria have been developed mainly to enroll appropriate subjects and exclude those with serious diseases or contraindications to treatment.

#### 2.2.1. Inclusion criteria

Participants diagnosed with subjective tinnitus^[1]^ and able to communicate normally in Chinese will be recruited in the study if they satisfied all of the following criteria: Males and females aged 50 to 70 years; Unilateral or bilateral tinnitus for 0.5 to 24 months; Loudness of tinnitus rated ≥ 3 points on a 0 to 10 numeric rating scale (NRS) at the time of enrollment; Agree to sign the informed consent form voluntarily.

#### 2.2.2. Exclusion criteria

Subjects will be excluded if they possess any of the following criteria: Known disease conditions that could cause tinnitus, including Meniere syndrome, external auditory canal diseases, and middle ear diseases; History of head trauma; Currently using a cardiac pacemaker or metal implants; Known severe cardiac diseases, cerebrovascular diseases, renal diseases, or hematologic diseases; Known severe psychiatric or psychological disorder; Pregnant, lactating, or expecting a pregnancy during the study period; Severe needle phobia; Known hypersensitive reaction following acupuncture and moxibustion treatment or an inability to cooperate with the acupuncture and moxibustion procedure; Incapable to understand and answer the questions of the assessors in the study; Other factors deemed unsuitable for inclusion in the study by investigators.

### 2.3. Recruitment procedure and study flow

**Figure 1.**
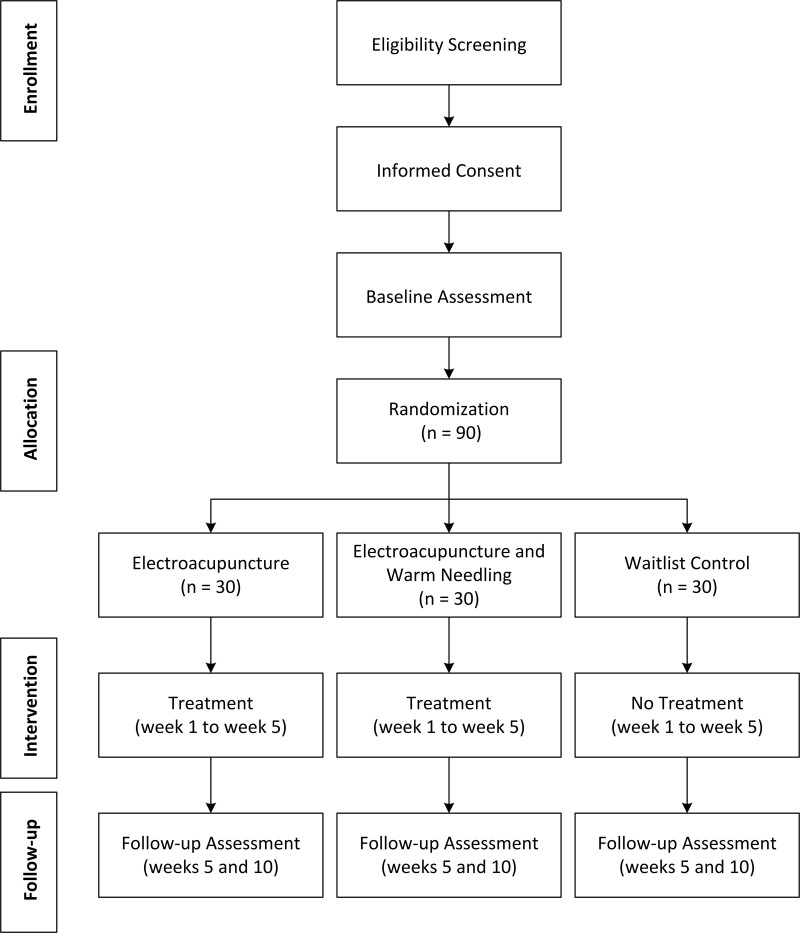
The study flowchart.

**Figure 2.**
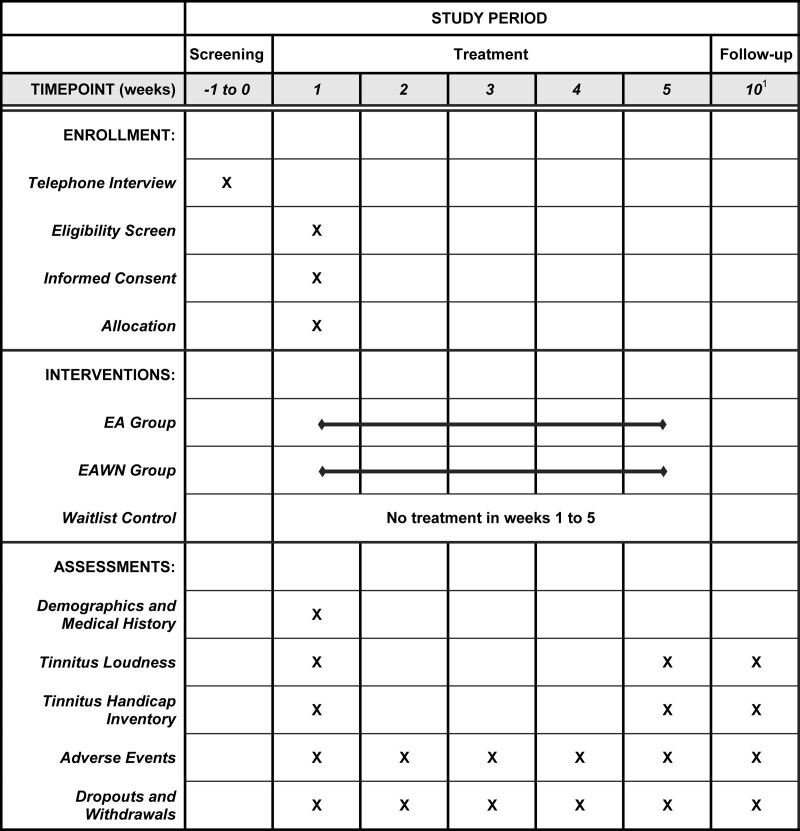
Schedule of enrollment, interventions, and assessments. ^1^ Visits for ± 4 days are allowed for week 10 follow-up assessments. EA = electroacupuncture, EAWN = electroacupuncture and warm needling.

Subjects will be recruited from all Chinese medicine centers, polyclinics, and mobile clinics under the management of Pok Oi Hospital. These sites cover all districts in Hong Kong. Subjects interested in participating in the study will be referred to or self-approached by the 3 dedicated Chinese medicine centers to undergo eligibility assessment. They will be prescreened through telephone interviews. Potential participants will undergo a face-to-face interview to confirm their eligibility for the study. During the interview, the investigators will explain the overall objectives and nature of the study, and undergo the eligibility assessment and informed consent process. A 4-week washout period will be required for those receiving acupuncture therapies before enrollment. If the subject consents, sociodemographic and clinical data, including sex, age, and medical history will be collected. Additionally, baseline assessments of primary and secondary outcome measures will be conducted. They will then be randomly assigned to 1 of the 3 groups by an independent staff member who is not involved in the study. A schedule for treatment and follow-up procedure will be assigned. After enrollment, the study site will strive to keep track of the participants throughout the study to maintain participation and complete follow-up assessments. The study flow is shown in Figure 1 and Figure 2 demonstrates the schedule of enrollment, interventions, and assessments.

### 2.4. Randomization and blinding

The treatment allocation of EA, EAWN, or waitlist control for each eligible subject in the study will be determined through a randomization process. Participants will be told that they will be randomized to 1 of 3 groups: EA for 5 weeks followed by observation for 5 weeks; EAWN for 5 weeks followed by observation for 5 weeks; or observation for 10 weeks followed by EA or EAWN for 5 weeks. Treatment or assessment will be scheduled to avoid contact between the participants. The random allocation to the 3 arms of the study will be generated using Random Allocation Software,^[17]^ following a balanced 1:1:1 ratio. A standard block size of 6 will be used, which will not be revealed to the site investigators. This will be performed by an independent administrator who is not involved in the study, and the information on the random allocation list will remain strictly confidential. The randomization result will be kept in sequentially numbered, opaque, sealed envelopes to ensure allocation concealment. In this study, practitioners and patients will not be blinded; however, the outcome assessors will be blinded to the intervention assignment.

### 2.5. Qualifications of practitioners

The treatments of the study will be conducted by Hong Kong registered Chinese medicine practitioners who have over 3 years of experience in acupuncture and are trained in administering EAWN treatment. The registered Chinese medicine practitioners in this study will strictly adhere to the study protocol and be trained in administering study treatments.

### 2.6. Treatment procedure

The style of acupuncture and moxibustion used in this study is based on TCM theory. Furthermore, the selection of acupuncture points and treatment regimens will be based on a review of the literature and previous studies,^[13,18]^ and expert opinion. The treatments will be provided free of cost to all the subjects.

#### 2.6.1. EAWN group

EAWN therapy will be performed twice per week for 5 weeks, for a total of 10 treatment sessions. During each treatment session, standardized procedures will be performed. For subjects with tinnitus on both sides, treatment will be provided bilaterally. The acupuncture points used will be Tinggong (SI19) and Yifeng (TE17) on the affected side; Baihui (GV20) and Yintang (GV24^+^); bilateral posterior temporal line (the scalp acupuncture line MS11); Zhongzhu (TE3) and Zusanli (ST36) on the affected side.^[19–21]^ Treatments will be administered using sterile, single-use, disposable acupuncture needles (0.25 mm × 25/40 mm; ARight Brand, Suzhou Medical Appliance Factory, Suzhou, China). Room temperature will be maintained at 25 ± 3^o^C during treatment. The subjects will lie down in a supine position during the treatment sessions. First, the skin will be sterilized before needle insertion. Subsequently, needles will be inserted in the following sequence: perpendicularly for SI19 (depth, 20 mm) and TE17 (depth, 20 mm); transversely for GV20 (depth, 15 mm), GV24^+^ (depth, 10 mm), and from Shuaigu (GB8) to Qubin (GB7) along MS11 (depth, 20 mm); perpendicularly for TE3 (depth, 10 mm) and ST36 (depth, 25 mm). Next, stimulation will be performed by twirling the needles evenly to achieve a dull ache, numbness, or heaviness sensation, referred to as deqi.^[22,23]^ After obtaining deqi, the tonifying method will be applied to ST36, and the neutral supplementation method will be applied to the remaining acupuncture points. All needles will be retained in situ for 25 minutes. During the retention period, electrical stimulation and warm needling will be performed simultaneously. Paired electrodes of the electroacupuncture apparatus (Hwato SDZ-III, Suzhou Medical Appliance Factory, Suzhou, China) will be connected to the needles at the ipsilateral SI19 and TE17, GV20 and GV24^+^, and bilateral MS11. Electrical stimulation will be delivered using dense-disperse waves at a frequency of 2 Hz. The intensity of the electric current will be increased to the patient’s maximum tolerance and then slightly reduced to a bearable and comfortable level (<10 mA). For warm needling, the bottom of a cylinder-shaped moxa stick (Changsha Furong Huai, Changsha, China) made of mugwort, measuring 11 mm in diameter and 15 mm in length, and weighting 0.7 ± 0.05 g will be ignited and attached to the needle handle (the needle handle is inserted into the center of the cylinder-shaped moxa stick) of SI19 and TE17. The ignited end of the moxa stick is positioned 35 mm from the skin surface, which is a safe and appropriate distance for the administration of moxibustion.^[24–27]^ After the first moxa stick burns out, the ashes will be removed, and the second stick will be placed following the same procedure. In total, 2 moxa sticks will be applied individually to each acupuncture point of SI19 and TE17. The burning time for each moxa stick is approximately 10 minutes. The total moxibustion time for 2 moxa sticks will be approximately 20 minutes, which is an advisable time for effective thermal stimulation of moxibustion.^[24,28]^ During warm needling therapy, the subject will feel a sense of local warmth, and the surrounding skin may become mildly red without any burning pain. The needles will be removed after the retention time and at the end of the moxibustion procedure.

During warm needling, precautionary measures should be taken. To prevent skin burns, the practitioner should hold the junction of the needle body with one hand and gently inserts the ignited moxa stick into the needle at the inserted acupuncture points with the other hand. After attaching the moxa stick to the needle handle, the practitioner should ensure that the stick is fully fixed to the needle handle to prevent it from falling onto the skin surface. When replacing the second moxa stick and removing the needles at the end of the treatment, the practitioner should carefully to check the temperature of the needles to prevent burns. The practitioner must be aware of the subject’s sensitivity to temperature and pain, and advise the subject not to let the acupuncture points become uncomfortably hot. The subject will be closely monitored during the entire procedure to ensure safety, and that any accident will be handled in a timely manner.^[29]^

#### 2.6.2. EA group

Subjects will receive EA treatment in the same manner as in the EAWN group. All treatment procedures for EA will be exactly the same as those used in the EAWN group, except that no warm needling therapy will be provided.

#### 2.6.3. Waitlist control group

No intervention will be provided for the waitlist control group, and the subjects in this group will be followed up during the 10-week observation period following the baseline assessment. In addition, the participants in this group will be assessed at weeks 5 and 10. After the 10-week waiting period, the participants in the control group will be re-randomized to receive free EAWN or EA treatments for tinnitus using the same treatment protocol that was administered to those in the EAWN or EA group. This is in consideration of the ethical implications of withholding potentially beneficial treatment during the 10-week waiting period.

### 2.7. Concomitant care and intervention

During the entire study period, each subject will be informed to avoid receiving other forms of intervention for tinnitus as they could influence the study results, including but not limited to other acupuncture treatments, behavioral therapy, and medication. Furthermore, the subjects will be advised by the practitioner to avoid other treatments, which will be reinforced by the investigators during the entry period and at each treatment session or evaluation time point. If a subject has attended other co-interventions for tinnitus, it should be reported to the investigators during each evaluation time, and the information will be recorded for analysis.

However, for basic diseases such as hypertension, diabetes, hyperlipidemia, coronary heart disease, and other complicated chronic diseases, subjects must continue taking their routine medication and other therapies. The research staff will record the names of the diseases, medications, and therapies in the case report form. This study will not provide posttrial care.

### 2.8. Study termination rules

The study may be terminated under the following circumstances: Participants experience serious adverse reactions or other complications that the investigators decide it is not safe to continue the study; Participants could not benefit or even get worse from the study treatment that the investigators consider necessary to terminate the study treatment; Participants who, for whatever reason, are unwilling or unable to continue the study and request the investigators to withdraw and discontinue the trial.

### 2.9. Outcome measurements

In this study, the unit of analysis is on an individual basis. Subjects with unilateral or bilateral tinnitus will be included without discrimination. Outcomes for tinnitus will be assessed at the side with more severe symptoms. Assessments and questionnaires will be administered at baseline (before intervention), week 5 (posttreatment), and week 10 (5-week follow-up).

#### 2.9.1. Primary outcome

Tinnitus loudness is a main symptom of tinnitus. The primary endpoint of our study will be the change in NRS score for tinnitus loudness from baseline to treatment completion (week 5). Changes in the mean NRS score for tinnitus from baseline to week 10 (5 weeks after treatment completion) will also be assessed. The NRS for the loudness of tinnitus is a widely used measurement for tinnitus.^[2]^ Subjects will rate the loudness of tinnitus using an 11-point NRS (0 − 10) with 0 indicating “no tinnitus”, and 10 indicating “maximum intolerable tinnitus”. The higher the score, the greater the tinnitus loudness and its effect.

#### 2.9.2. Secondary outcome

Tinnitus handicap inventory (THI) will be used as the secondary outcome measurement. The THI is a commonly used self-report measure for evaluating the extent of tinnitus-related distress with good reliability and validity.^[30]^ In this study, the validated Chinese (Cantonese) version of the THI will be adopted.^[31]^ This questionnaire consists of 25 items and can be categorized into 3 subscales: functional (12 items), emotional (8 items), and catastrophic (5 items). Each item will be scored on a 3-point rating scale. A yes will score 4 points; sometimes, 2 points; and no, zero points. The total THI score range from 0 to 100, with a higher score indicating a greater impact of tinnitus on the patients quality of life.

### 2.10. Safety assessment

Subjects will be asked to report any adverse events. Adverse events related to acupuncture and moxibustion include pain, bruising, bleeding, burns, blisters, dizziness, anxiety, and infections.^[32,33]^ For safety concerns and evaluations, all unexpected and unintended responses that are not necessarily related to the study treatment will be recorded with a detailed explanation including time of occurrence, duration of symptoms, seriousness (mild, moderate, or severe),^[32]^ management measures, time of adverse reaction disappearance, and causality categorization (certain, probable or likely, possible, unlikely, unclassified, or unclassifiable)^[34]^ at every visit on an adverse event form. Site investigators will record and deal with all adverse events regardless of whether they are relevant to the study treatment.

### 2.11. Dropouts and withdrawals

The participants are able to withdraw from the study for any reason at any time. All dropouts and attritions during the course of the study will be monitored and recorded with respective reasons.

### 2.12. Data collection and management

All participants’ data will be collected after obtaining signed consent forms. The participant’s privacy will be strictly protected throughout the trial. Investigators are responsible for maintaining the anonymity of the subjects. Participants will be assigned code numbers as identifiers and their confidentiality will be protected by the study identification number. The collected data will be anonymized and recorded on case report forms. All paper-based data will be checked and input into a password-protected electronic database. Two data entry persons will enter all data into an electronic database by double data entry to ensure data accuracy. All data will be kept confidential and access to the data will be limited to delegated research personnel. The participants study information will not be released outside the study without obtaining written permission from the participant. After the trial, all documents will be preserved in secured research archives for 7 years and will be deleted after the completion of the storage period.

### 2.13. Statistical analysis

#### 2.13.1. Sample size calculation

The sample size calculation will be based on the primary outcome measure of the NRS score of the loudness of tinnitus (0 − 10 points) and performed using OpenEpi.^[35]^ An estimation of the between-group difference and standard deviation is necessary because of the lack of similar comparable studies. In this study, we assume that similar differences in the NRS score of tinnitus loudness will be detected between the EA and waitlist control groups or the EAWN and waitlist control groups. We presume that a clinically significant difference of mean 2-point reduction and standard deviation of 2.49 in the NRS score of tinnitus loudness is detected based on previous studies^[36,37]^ and expert opinion. Using a 2-sided test, an alpha value of 0.05, and a power of 80%, it is estimated that 25 subjects will be required per study group. Considering a 20% attrition rate, the sample size for each group will be 30 subjects, which implies a total sample size of 90 subjects for the entire trial.

#### 2.13.2. Statistical method and analysis

Statistical analysis of the collected data will be performed using IBM SPSS Statistics 27 (IBM Corp, Armonk, USA). An intention-to-treat approach will be used in this study. All randomized subjects will be included in the analysis, regardless of whether they received treatment. In addition, interim analyses will not be performed.

Descriptive statistics will be used to summarize participants baseline characteristics by group using mean (standard deviation), median (interquartile range), or number (percentage) as appropriate. All measurement data will first be assessed using normality tests. One-way analysis of variance or Welch analysis of variance will be used for comparison among the 3 groups for continuous variables. Categorical variables will be compared using the Pearson chi-squared test (or Fisher exact test).

Analyses of the primary and secondary continuous outcome variables among the 3 study groups (EA, EAWN, and waitlist control) overtime (baseline, weeks 5 and 10) will be performed using linear mixed models for repeated measures analysis, with treatment, time point, and interaction between treatment and time point included as fixed effects, within-person correlation modeled as a random effect, and age and tinnitus duration adjusted as covariates in the model. Missing data will be processed using the mixed effects model, assuming missing at random. Significant results (significant interaction, time × study groups) will be subject to post hoc tests to determine which group scores differently. Mean differences at weeks 5 and 10 will be estimated by the treatment × time interaction term, with associated 95% confidence intervals and *P* values.

Comparisons between the EA and EAWN groups for the adverse event rate among the 3 groups will be evaluated using the Pearson chi-squared test (or Fisher exact test).

The results will be considered statistically significant at a probability level of *P* < .05 with a 2-sided test throughout the analysis. In post hoc tests, we will employ the Bonferroni correction method and appropriately adjust the overall level of significance for multiple primary and secondary outcomes at different time points.

### 2.14. Study monitoring and quality control

To guarantee the quality of the study, advance training will be carried out before subject recruitment to ensure that all investigators and related staffs adhere to the study protocol. The investigators will assure that all reported trial data are accurate, complete, and verifiable from the source documents. To oversee the progress of the study, quarterly monitoring meetings will be held between the investigators and the Hospital Authority Chinese Medicine Department, which is completely uninvolved in the running of the trial and has no competing interests.

### 2.15. Patient and public involvement

Participants and the public will not be involved in the design, development, recruitment, or conduct of this clinical trial.

### 2.16. Ethical consideration and dissemination plans

The study will be conducted in compliance with local law and will be carried out in accordance with the ethical principles of the Declaration of Helsinki^[38,39]^ and the ICH-GCP guidelines. Prior ethical approval (CREC Ref. No. 2022.327-T) has been obtained from the Joint Chinese University of Hong Kong − New Territories East Cluster Clinical Research Ethics Review Committee before the commencement of the study. Any changes to the protocol will be reported to this committee, and amendment approval should be obtained before any changes can occur. Each participant will voluntarily sign an informed consent form before participation. This study is registered at ClinicalTrials.gov (Identifier: NCT05557357). We plan to publish the results of this clinical trial in a peer-reviewed medical journal or academic conference proceedings.

## 3. Discussion

Tinnitus is a condition characterized by the perception of a persistent or intermittent sound without an external sound source. It is an invisible condition and a common otologic disorder that can be exasperated. It is a burdensome condition at both individual and societal levels. However, tinnitus treatment remains challenging.

Acupuncture and moxibustion have been used in otorhinolaryngology to treat tinnitus for thousands of years by regulating the flow of *qi* and blood in the body.^[10]^ A previous study reported that acupuncture can cause an effect on the cochlear function in patients with tinnitus, with a significant change in the amplitude of otoacoustic emissions.^[40]^ It can also affect the contralateral ear through the medial olivocochlear system, which may lead to tinnitus regression. Moreover, through needling in the periauricular region, the effect of acupuncture on tinnitus was associated with an improvement in cochlear blood flow via the infrared thermography test.^[41]^ In addition, acupuncture may have an effect on tinnitus by adjusting the central neurotransmitters gamma-aminobutyric acid and 5-hydroxytryptamine content.^[42]^ However, due to the heterogeneity of previous studies and limitations in trials with high levels of evidence, positive findings cannot be generalized, and the results are not conclusive.^[11,14]^

This study is a rigorously designed evidence-based clinical trial to evaluate whether EA or EAWN has therapeutic effects in reducing tinnitus loudness and improving tinnitus disability. The trial focus on 50 to 70 years old patients as these age groups have a higher prevalence of tinnitus.^[5]^ Carefully designed treatment procedures are developed, and proper training will be provided to participating practitioners for standardization of intervention for both treatment groups. Besides, validated and frequently used outcome measures of the NRS and THI will be used.^[43]^ Loud tinnitus is one of the most frequent clinical complaints of patients with tinnitus. NRS is commonly used to provide numeric estimates of tinnitus loudness. Subjective loudness can simply and quickly measure loudness and distress in patients with tinnitus^[44]^ and will be set as the primary outcome measure. In addition, the THI is a validated and specific questionnaire that measures the impact of tinnitus on daily life and symptom severity. Moreover, the THI has been proven to yield excellent internal consistency and reliability, and significant correlations have been found between the THI and symptom rating scales.^[30]^ By using these validated instruments, proper treatment results will be generated.

Nevertheless, this study has some limitations. Owing to the nature of the intervention, it is difficult to develop proper blinding for participants and practitioners on electroacupuncture and warm needling, which may lead to a high risk of performance bias. Moreover, the use of self-reported and subjective assessment tools may introduce bias or variability in the outcome measures. In addition, limited resources will only allow for 5-week posttreatment follow-up assessments, and the longer-term effects of the 2 interventions will not be evaluated.

## 4. Conclusion

This protocol describes the details of a randomized controlled clinical trial for tinnitus. The findings from this proposed randomized controlled trial will provide additional clinical evidence on whether EA or EAWN could be of value for the management of tinnitus among older adult patients, and facilitate clinical practice and scientific studies in the future.

## Acknowledgements

The authors give special thanks to all the patients for their participation in the study. The authors also gratefully thank all the research team members for their contributions to the implementation of the trial.

## Author contributions

**Conceptualization:** Yuanqi Guo, Lai Fun Ho, Zhi Xiu Lin.

**Data curation:** Yuanqi Guo, Lai Fun Ho.

**Formal analysis:** Yuanqi Guo, Lai Fun Ho, Zhi Xiu Lin.

**Funding acquisition:** Yuanqi Guo, Min Chen.

**Investigation:** Yuanqi Guo, Lai Fun Ho, Liyi Chen, Ping Him Tsang.

**Methodology:** Yuanqi Guo, Lai Fun Ho, Liyi Chen, Ping Him Tsang, Kam Leung Chan, Bacon Fung-Leung Ng, Zhi Xiu Lin.

**Project administration:** Lai Fun Ho, Kam Leung Chan.

**Resources:** Lai Fun Ho, Ping Him Tsang.

**Supervision:** Yuanqi Guo, Min Chen, Bacon Fung-Leung Ng, Zhi Xiu Lin.

**Writing – original draft:** Lai Fun Ho.

**Writing – review & editing:** Yuanqi Guo, Lai Fun Ho, Liyi Chen, Ping Him Tsang, Min Chen, Kam Leung Chan, Bacon Fung-Leung Ng, Zhi Xiu Lin.
